# Placental Transmogrification of the Lung Presenting as Cystic‐Solid Mass: A Case Report

**DOI:** 10.1111/crj.13807

**Published:** 2024-07-12

**Authors:** Chen Liu, Yunhui Zhang, Dengyuan Li, Danxiong Sun

**Affiliations:** ^1^ Department of Respiratory and Critical Care Medicine The First People's Hospital of Yunnan Province Kunming China; ^2^ Faculty of Life Science and Technology Kunming University of Science and Technology Kunming China

**Keywords:** cystic‐solid mass, placenta‐like villous structures, placental transmogrification of the lung, pulmonary cystic lesion

## Abstract

The gradually progressive solitary cystic‐solid mass of chest CT scans is highly suggestive of lung cancer. We report a case of a 29‐year‐old woman with a persistent cystic‐solid lesion in the right upper lobe. A chest CT scan showed a 35 mm × 44 mm × 51 mm focal cystic‐solid mass in the anterior segment of the right upper lobe. The size of lesion had increased over 3 years, especially for the solid component. The right upper lobe pneumonectomy was performed. Postoperative pathological examination showed placental transmogrification of the lung, which is a rare cause of pulmonary cystic lesion.

## Graphical abstract

The chest CT scan showed a 35 mm × 44 mm × 51 mm focal cystic‐solid mass in the anterior segment of the right upper lobe which suggested a presumed diagnosis of cystic lung cancer. Postoperative pathological examination showed placental transmogrification of the lung.

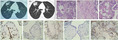

## 1 Introduction

Infection, inflammation, non‐infectious granulomatous disease, and lung cancer can all present with pulmonary cystic‐solid mass on chest radiographs. But the gradually progressive solitary cystic‐solid mass of chest CT scans is highly suggestive of lung cancer. We report a case of a young woman with a progressive cystic‐solid lesion in the right upper lobe, mimicking cystic lung cancer. Postoperative pathological examination showed placental transmogrification of the lung (PTL). To our knowledge, few authors had mentioned PTL as one of the differential diagnoses of cystic lung cancer in the previous literatures.

## 2 Case Presentation

**FIGURE 1:**
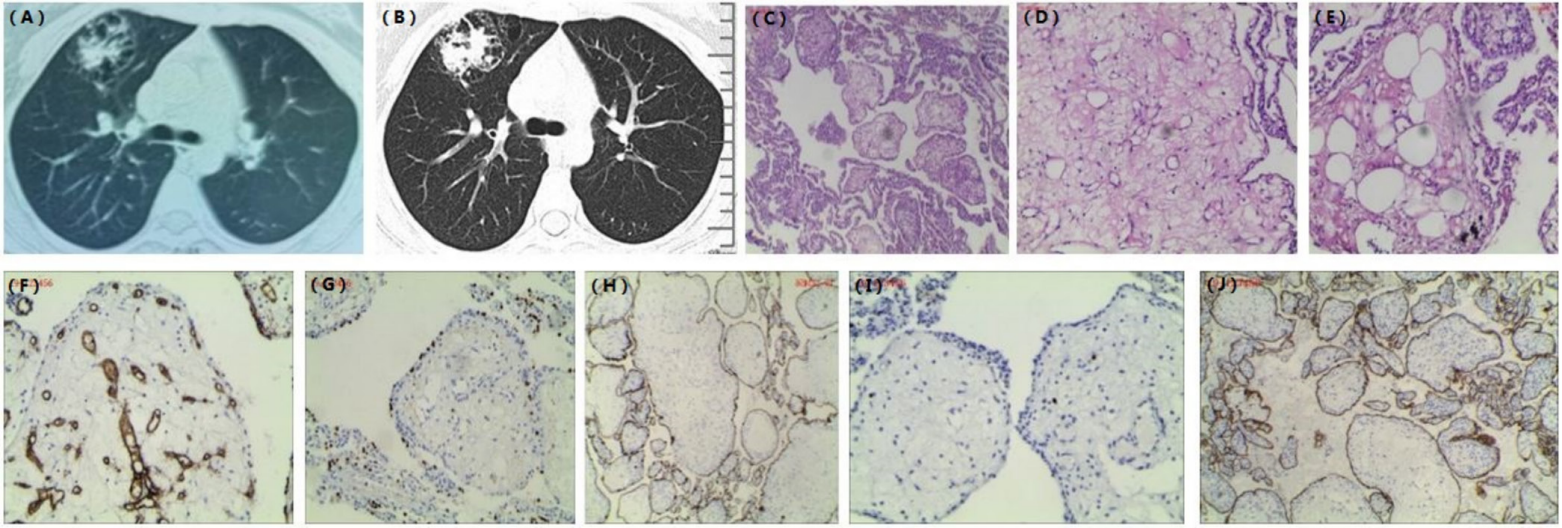
(A) Three years ago, the chest CT revealed a cystic‐solid mass localized to the right upper lobe. (B) The chest CT scan showed a 35 mm × 44 mm × 51 mm focal cystic‐solid mass in the anterior segment of the right upper lobe. The size of lesion had increased over 3 years, especially for the solid component. (C) Histopathologic examination showing placental villus‐like papillary structures. (D) The papillary cores contained dilated capillaries. (E) Adipocytes were observed in the focal papillary structures. (F) The central capillaries of the loose fibrous vascular axis were positive for CD34. (G) TTF‐1 were partly positive. (H) The lining cells were positive for PCK. (I) A few cells in the center of the fibrous capillaries were positive for ki‐67. (J) PCK(+), CK7(+).

A 29‐year‐old woman was admitted to our respiratory department due to a gradually progressing cystic‐solid mass localized to the right upper lobe. Three years ago, she was examined following incidental observation of an unsuspected mass on a chest X‐ray in a local hospital. Computed tomography scan revealed a cystic‐solid mass localized to the right upper lobe (Figure 1A). She had no complaints of cough, sputum, or shortness of breath. She was hospitalized in a different facility with a presumed diagnosis of pulmonary tuberculosis and treated empirically with anti‐tuberculosis drugs for 1 year. However, radiologic abnormalities persisted. Two months ago, the patient developed cough and phlegm. A repeat chest CT demonstrated a progressive cystic‐solid mass. There was no significant past medical history. She was asymptomatic at the current visit. The patient's physical examination findings were within normal range.

Laboratory investigations revealed her complete blood count, liver function, and kidney function were normal. The β(1,3)‐d‐glucan was 180.86 pg/mL (normal, < 70 pg/mL). The percentage of eosinophil count in induced sputum was 3.5% with a normal eosinophil count in blood. The carbohydrate antigen 125 level was 82 U/mL (normal, 0–35 U/mL), the rest of the tumor markers (alpha fetoprotein, carcinoembryonic antigen, neuron‐specific enolase, squamous cell carcinoma antigen, cytokeratin 19 fragment, carbohydrate antigen 153, carbohydrate antigen 199, and carbohydrate antigen 724) were all within normal ranges. The whole blood IFN‐γ release assay was positive (0.39 IU/mL; normal level, < 0.35 IU/mL). Spirometry was normal (the FEV1%pred, FVC%pred, FEV1/FVC, and TLC%pred were 80.5%, 85.3%, 82.0%, and 87.7%, respectively). Tracheobronchial tree inspection results by flexible video bronchoscopy were unremarkable, and BAL revealed values of 3.3% eosinophils. Results of the acid‐fast bacilli smear were negative. The smear and culture of BALF for fungi, acid‐fast bacilli, and other bacteria were all negative. Malignant cells were not observed.

The chest CT scan showed a 35 mm × 44 mm × 51 mm focal cystic‐solid mass in the anterior segment of the right upper lobe. The size of lesion had increased over 3 years, especially for the solid component (Figure 1B). The chest CT suggested a presumed diagnosis of cystic lung cancer. In order to elucidate the underlying pathology, the right upper lobe pneumonectomy was performed. Postoperative pathology reported inflammatory pseudotumor.

Because pathological diagnosis did not match clinical manifestations of the patient, the specimen was sent to Peking Union Medical College Hospital. Microscopic examination revealed multiple placental villus‐like papillary structures, and adipocytes were observed in the focal papillary structures (Figure 1C–E). The papillary cores contained dilated capillaries. Immunohistochemical examination showed the placental villus‐like papillary structure was lined with epithelium and PCK(+), CK7(+), and TTF‐1 (partial+) (Figure 1G,J). The capillaries in the loose fibrous vascular axis were positive for CD34, and a few cells in the center of the fibrous capillaries were positive for ki‐67 (Figure 1F,I). The pathological consultation opinion was PTL

The patient had an uneventful clinical course after surgery and was discharged without symptoms. At 1‐year follow‐up, repeat CT did not demonstrate recurrence of disease.

## 3 Discuss

PTL is a rare benign lesion that microscopically resembles immature placental tissue, with formation of placental villus‐like papillary structures covered by epithelial cells, although they do not carry any biological and biochemical features of a placenta. It was first described by McChesney in 1979 [1]. So far, the source and pathogenesis of PTL are unidentified. The major hypotheses include hamartomatous malformation, lymphovascular proliferation in the setting of an emphysematous lung, and clonal hyperplasia of stromal cells, but none of these are based on sound morphologic or biological evidence. PTL may represent a benign incidental morphologic transformation rather than an independent disease in some authors' opinion [2].

Being extremely rare, the PTL is unknown to major pathologists and chest doctors. The diagnosis is easily missed or ignored in many cases, which could be a cause of the low reported prevalence of the condition. As a tertiary care university hospital, the pathologists misdiagnose PTL as inflammatory pseudotumor in our case, indicating that there may be missed diagnosis in a large number of PTL cases in community hospitals. When the clinical diagnosis of PTL is suspected, good communication between the surgeon and the pathologist should be encouraged. Ortiz and Tortosa [3] presented a retrospective study of all cases of bullous emphysema (410) and pulmonary hamartoma (103) diagnosed in a reference center between 2000 and 2015. Placental transmogrification was identified in 3 of 513 cases (0.58%). Yaprak Bayrak, Vural, and Yildiz [4] retrospectively reviewed histologic slides of 35 cases of pulmonary hamartomas between 2001 and 2021. Five of these patients (17.9%) had pulmonary placental transmogrification components in resection materials with varying degree between 5% and 80%.

The age of presentation varied from 13 to 72 years, but PTL is most common in men between 20 to 50 years of age [5, 6]. Clinically, PTL is either asymptomatic or presented with cough, hemoptysis, chest pain, dyspnea, pneumothorax (possibly tension pneumothorax), or a combination of all of the above. A bullous emphysema pattern with a mixed pattern of thin‐walled cystic lesions and nodules is the most common radiological findings of PTL. In a few cases, it masqueraded as pneumonia or interstitial lung disease or associated with lung adenocarcinoma or unilateral pleural effusion [7, 8, 9, 10].

Differential diagnosis of PTL includes bullous emphysema, hamartoma, congenital cystic adenomatoid malformation, bronchogenic cyst, cystic lung tumors, and intralobar pulmonary sequestration. Due to the lack of characteristic radiological manifestations, the definitive diagnosis of PTL requires pathological analysis. Grossly and microscopically, the characteristic pathological change is that the lesion resembles placental tissue, with formation of placental villus‐like papillary structures covered by epithelial cells [6]. In our case, the radiological manifestation was solitary cystic‐solid mass of which differential diagnosis mainly refers to other cystic lung diseases, such as cystic lung cancer, congenital cystic adenomatoid malformation, and infectious diseases (especially pulmonary mycosis). Cystic lung cancer usually presents as progressive cyst wall thickening or nodularity abutting a cystic airspace [11]. Although the solid internal component gradually progressed over time, unlike typical cystic lung cancer, the cyst wall did not thicken in our case, which may help differentiate it from cancer. Pulmonary acute infection (especially fungal infections) also can cause large cystic‐solid mass in the form of mycetomas. The patient had a long disease course and no significant infection symptoms, which were not consistent with infectious diseases. It is unlikely to distinguish between PTL and congenital cystic adenomatoid malformation from radiological imaging, and in addition, congenital cystic adenomatoid malformation also can be present in adults.

Given that PTL may progress and result in severe pulmonary symptoms, and there was no literature report of recurrence in those in whom complete resection was achieved, surgical resection is the preferred treatment. To avoid the lobectomy and pneumonectomy, almost all authors recommend that these pathologic lesions are best treated by minimal resection with preservation of healthy lung tissue if at all possible. In our case, if PTL had been suspected before surgery, it was possible to avoid lobectomy, leaving as much normal lung tissue as feasible.

PTL should be one of the differential diagnoses for cystic‐solid mass. The thin walled cystic‐solid masses may indicate the diagnosis of PTL. Here, we sought to increase the awareness of PTL among pathologists and pulmonologists and highlight the importance of a preoperative suspected diagnosis for pulmonologists which could facilitate minimal resection surgery and preserve more normal lung tissue.

## Author Contributions

Data acquisition: Chen Liu. All authors contributed to the writing and revision of this manuscript.

## Ethics Statement

Appropriate written informed consent was obtained from the patient for the publication of this case report and accompanying images. It was approved by the Clinical Research Ethics Committee of the First People‘s Hospital of Yunnan Province and was implemented.

## Conflicts of Interest

The authors declare no conflicts of interest.

## Data Availability

The data that support the findings of this study are available from the corresponding author upon reasonable request.

## References

[1] T. M. McChesney , “Placental Transmogrification of the Lung: A Unique Case With Remarkable Histopathologic Features,” Laboratory Investigation 40 (1979): 245–246.

[2] M. Yang , X. T. Zhang , X. F. Liu , and X. Y. Lin , “Placental Transmogrification of the Lung Presenting as a Peripheral Solitary Nodule in a Male With the History of Trauma: A Case Report,” Medicine (Baltimore) 97, no. 18 (2018): e0661, 10.1097/MD.0000000000010661. 29718888 PMC6392622

[3] S. Ortiz and F. Tortosa , “Pulmonary Placental Transmogrification: The Last 16 Years in a Reference Centre,” Revista Portuguesa de Pneumologia 23, no. 3 (2017): 164–166, 10.1016/j.rppnen.2017.02.007. 28372973

[4] B. Yaprak Bayrak , C. Vural , and K. Yildiz , “Pulmonary Placental Transmogrification: A Difficult Pattern in Differential Diagnosis of Pulmonary Hamartomas From a Tertiary Care Hospital in Turkey,” Journal of Cardiothoracic Surgery 18, no. 1 (2023): 127, 10.1186/s13019-023-02217-1. 37041644 PMC10091638

[5] B. Özsezen , U. Özçelik , D. Doğru , et al., “A Child Presenting With Bullous Emphysema,” The Turkish Journal of Pediatrics 64, no. 5 (2022): 964–969, 10.24953/turkjped.2021.5515. 36305451

[6] D. J. Ma , H. S. Liu , S. Q. Li , et al., “Placental Transmogrification of the Lung: Case Report and Systematic Review of the Literature,” Medicine (Baltimore) 96, no. 35 (2017): e7733, 10.1097/MD.0000000000007733. 28858088 PMC5585482

[7] K. Horiuchi , T. Asakura , S. Sakaguchi , F. Saito , and J. Yamamoto , “Placental Transmogrification of the Lung Masquerading as Difficult‐To‐Treat Pneumonia,” QJM 113, no. 3 (2019): 213–214, 10.1093/qjmed/hcz153. 31225601

[8] A. Lohbrunner , L. Bitton , J. Remy , B. Wallaert , and C. Chenivesse , “Lung Placental Transmogrification Presenting as Progressive Unilateral Interstitial Lung Disease,” Sarcoidosis, Vasculitis, and Diffuse Lung Diseases 34, no. 2 (2017): 188–190, 10.36141/svdld.v34i2.5236. PMC7170147 32476842

[9] A. Hamza , S. Khawar , M. S. Khurram , et al., “Pulmonary Placental Transmogrification Associated With Adenocarcinoma of the Lung: A Case Report With a Comprehensive Review of the Literature,” Autopsy & Case Reports 7, no. 3 (2017): 44–49, 10.4322/acr.2017.027. 29043210 PMC5634434

[10] N. Narula , S. Ngu , D. Sharma , F. Siddiqui , and M. Chalhoub , “Placental Transmogrification of the Lung Associated With Unilateral Pleural Effusion: A Case Report With a Comprehensive Review of the Literature,” Respiratory Medicine Case Reports 26 (2019): 161–164, 10.1016/j.rmcr.2018.11.018. 30622891 PMC6319187

[11] A. Snoeckx , P. Reyntiens , L. Carp , et al., “Diagnostic and Clinical Features of Lung Cancer Associated With Cystic Airspaces,” Journal of Thoracic Disease 11, no. 3 (2019): 987–1004, 10.21037/jtd.2019.02.91. 31019789 PMC6462709

